# Influence of Elevated Preovulatory Progesterone on the Outcome of In Vitro Fertilization

**DOI:** 10.7759/cureus.96896

**Published:** 2025-11-15

**Authors:** Emina Ejubovic, Miro Kasum, Asim Spahovic, Jasmin Hodzic, Aida Hajdarpasic, Emir Mahmutbegovic

**Affiliations:** 1 Department of Gynaecology, Obstetrics and Reproductive Medicine, Sarajevo Medical School, Sarajevo School of Science and Technology, Sarajevo, BIH; 2 Department of Gynaecology and Obstetrics, University Hospital Centre Zagreb, University of Zagreb, Zagreb, HRV; 3 Department of Gynaecology and Obstetrics, General Hospital "Prim.dr.Abdulah Nakas", Sarajevo, BIH; 4 Department of Gynaecology and Obstetrics, School of Medicine, University of Zenica, Zenica, BIH; 5 Department of Medical Biology and Genetics, Sarajevo Medical School, Sarajevo School of Science and Technology, Sarajevo, BIH; 6 Department of Gynaecology and Obstetrics, Sarajevo Medical School, Sarajevo School of Science and Technology, Sarajevo, BIH

**Keywords:** in vitro fertilization, ivf, ivf outcome, preovulatory progesterone, progesterone

## Abstract

Aim: This study aims to investigate the influence of elevated preovulatory progesterone (P4) on the outcome of in vitro fertilization (IVF).

Materials and methods: This was a prospective cohort study conducted at the Clinic for Women's Diseases and Childbirth in Petrova, Zagreb, Croatia, in the period 2015-2017, which included a total number of 400 patients (300 in the control group and 100 in the group with elevated preovulatory P4). The study group was furthermore divided into normal responders (85 patients) and high responders (15 patients). The IVF outcome among patients with normal preovulatory P4 as compared to the patients with elevated preovulatory P4, the IVF outcome among normal and high responders, the influence of the embryo transfer type - blastocyst embryo transfer (BET) or frozen/thawed embryo transfer (FET) - among patients with elevated preovulatory P4 on the outcome of IVF procedures has been investigated in this study.

Results: Positive IVF outcome has been recorded in about one third of all the performed procedures in the control and the study group (P=0.762 for the conception rate and P=0.245 for the clinical pregnancy rate), with slightly better outcome with elevated preovulatory P4. A better outcome with FET has been observed among normal responders (P=0.019), and a slightly better outcome with BET has been noted among high responders (P=0.622).

Conclusion: Elevated preovulatory P4 has a slightly protective effect on IVF outcome, while the P4 on the fifth day >425.7 nmol/L could be a potential marker for the success of the IVF procedures among all the patients, and with P4 on the fifth day >384.6 nmol/L among patients with elevated preovulatory P4.

## Introduction

In the menstrual cycle, functional luteolysis is characterized by the loss of the ability of the corpus luteum to produce progesterone (P4), and normally, P4 values ​​reach their lowest point (<1.5 nmol/L) during the early follicular phase [1]. It has been demonstrated that the main secretory source of P4 in the early follicular phase is the adrenal gland, while the ovaries represent the main source of circulating P4 in the late follicular phase [2]. The clinical significance of elevated P4 values ​​in the early follicular phase of the menstrual cycle on days 4 and 5 of the cycle is considered to be the result of inadequate luteolysis. As it is present in 11.3% of infertile patients, it has been suggested that it may negatively affect fecundation [3].

Using a short protocol with gonadotropin-releasing hormone (GnRH) and high-dose follicle-stimulating hormone (FSH) in patients with a poor response to induction, 85% of cycles were exposed to an increase in P4 (>3.18 nmol/L) between cycle days 2 and 6 [4]. In a later study, in which a short protocol with GnRH was used, 32% of patients had elevated basal P4 values ​​(>3.18 nmol/L) on the fourth day of stimulation [5]. In cycles with GnRH using a long protocol, measurable P4 values ​​(>0.9858 nmol/L) were shown to be associated with a higher dropout rate and a lower fertilization rate with elevated P4 values ​​(0.954-<3.18 nmol/L) on the fifth day of stimulation [6]. In patients who were treated with GnRH antagonists (GnRH-ant) and FSH in the in vitro fertilization (IVF) procedure, in 4.9% of cycles, elevated P4 values ​​were present on the second day of the cycle, and it was concluded that the presence of elevated P4 values ​​is associated with a reduced possibility of pregnancy [7].

An increase in preovulatory P4 during ovulation induction, before the introduction of GnRH analogues, was often present in the late follicular phase when human chorionic gonadotropin (hCG) was administered to women in the IVF procedure (“premature luteinization”). With the introduction and use of GnRH analogues during the induction of ovulation with gonadotropins in the IVF procedure, it was thought that the analogues would prevent the rise of premature LH and preovulatory P4 on the day of hCG injection administration [8]. Nevertheless, despite the fact that premature luteinizing hormone (LH) surge is prevented by GnRH analogs during ovulation induction, a moderate P4 surge still occurs in 12.4 to 52.3% of cases.

Application of relatively high doses of FSH is necessary to achieve multifollicular growth, and it has been shown that the premature increase of preovulatory P4 could be affected by higher doses of FSH. In this way, the conversion of pregnenolone into P4 was increased, and thus the value of preovulatory P4 [9]. The role of preovulatory P4 in terms of its influence on the outcome of IVF has not yet been fully clarified and has recently been the subject of numerous studies and discussions [9,10].

The uneven discrimination limit between normal and elevated P4 values, which ranges widely between 2.5 and 7 nmol/L, although the most commonly used value is 4.77 nmol/L, also contributes to the disagreement [11,12]. One group of researchers believes that an elevated concentration of preovulatory P4 (>1.5 ng/ mL vs. 4.77 nmol/L) leads to an accelerated secretory transformation of the endometrium, weaker receptivity, and shorter implantation time, which adversely affects the outcome of IVF pregnancy, with a lower conception rate and clinical pregnancy rate [13-15]. Contrary, other researchers did not find an adverse effect of elevated preovulatory P4 concentrations (3 to 7 nmol/L) on the outcome of pregnancy after the IVF procedure compared to patients with normal P4 levels [16,17]. Some authors highlight people who respond well to ovulation induction (high responder, E2>3000 pg/mL, number of oocytes obtained >20), in whom the elevated concentration of preovulatory P4 (>5,724 nmol/L) is not significantly important for the outcome of pregnancy after an IVF procedure [18,19]. That is why the hypothesis in this research was to establish whether the elevated preovulatory P4 among normal and high responders has a negative effect on the IVF outcome.

## Materials and methods

This research is a prospective cohort study conducted in the Clinic for Women's Diseases and Childbirth, Petrova, Zagreb, Croatia, in the period 2015-2017. Patients in whom P4 is elevated on the second, third, and fourth days of the cycle, women older than 37 years, and poor responder patients (number of follicles <4 on hCG day, E2<500 pg/mL) were excluded from the study because each of the mentioned parameters adversely affects the outcome of the IVF procedure. Furthermore, patients who underwent intrauterine insemination (IUI) or who used anti-estrogens (clomiphene citrate (CC) and letrozole) in the IVF procedure, as well as patients undergoing the same procedure to preserve fertility due to malignant disease, were also excluded from the study. All other patients undergoing an IVF procedure were included in the research after signing the informed consent and after the approval of the Ethical Committee.

A total of 400 stimulated patients in the IVF procedure were prospectively analyzed by determining P4 and E2 on the day of hCG administration. Patients with normal preovulatory P4 values ​​(<4.77 nmol/L) represented the control group (300 women), while patients with elevated P4 values ​​(>4.77 nmol/L) represented the study group (100 women). The study group was further divided into two subgroups; the first group (85 women) was represented by normal responder patients, while the second group (15 women) was represented by high responder patients. All subjects were matched according to age, body mass index (BMI), stimulation protocol, and doses of gonadotropin, and the number of aspirated oocytes and transferred embryos. All patients who, after being informed about the potentially unfavorable influence of elevated P4 values ​​on the outcome of IVF, agreed to the research, were included in the study after signing the written consent.

Ovulation stimulation in patients was performed with GnRH analogues in combination with gonadotropins, and their dose and selection were adjusted according to the patient's age, FSH value, antral follicle count (AFC), anti-Mullerian hormone (AMH), BMI, and previous IVF procedure.

Due to the greater ovarian reserve in younger patients, lower doses of medication for ovulation stimulation were needed as compared to the older patients. In patients with polycystic ovaries, preparations containing a dominant FSH and a minimal concentration of LH were used. In cases where there was a risk of ovarian hyperstimulation syndrome (OHSS), milder stimulation protocols with gonadotropins in combination with GnRH antagonists and GnRH agonists for ovulation induction were applied. Medicines used for ovulation stimulation and induction: follitropin alfa, follitropin beta, triptorelin, cetrorelix acetate, choriogonadotropin, and menotropin.

Folliculogenesis was monitored by ultrasound and the E2 determination. When more than three follicles with a diameter of 17 mm or more were detected on the ovaries by the ultrasound, hCG was administered, and 36 hours later, a transvaginal aspiration of the oocytes of each follicle with a diameter greater than 17 mm was performed. Atrophic and degenerated oocytes were excluded from further procedure.

The level of P4 and the ratio of P4/E2 on the day of hCG were determined from the blood by an automated immunochemiluminescence method, while the men's sperm were analyzed and prepared in the laboratory, after which fertilization was performed by intracytoplasmic sperm injection (ICSI). Based on the E2 value and the number of oocytes obtained, a group of normal responder subjects (E2<3000 pg/mL, number of oocytes >20) and high responder subjects (E2>3000 pg/mL, number of oocytes >20) were observed separately.

In all patients, luteal support was provided by administering micronized P4 tablets vaginally, which the study subjects used for 12 days in a daily dose of 600 mg (three doses of 200 mg each). Since all subjects received micronized P4 after aspiration, it did not affect the results of the preovulatory P4 study. Given that different populations have different levels of P4 after ovulation on day 5, the mean value of P4 on day 5 was determined in the control group, which compared the discrimination limit based on which the patients of the study group were classified for frozen/thawed embryo transfer (FET) or blastocyst embryo transfer (BET). In the study group, FET was performed for normal responders with higher P4 values ​​on the fifth day than the discrimination limit, and BET was used for lower values. In high responders with elevated preovulatory and postovulatory P4 values, a randomized fresh or delayed BET was performed.

Primary study objectives included hormonally determined pregnancy or conception rate and clinical pregnancy rate. Hormonally confirmed pregnancy was defined as a positive value of beta-hCG 14 days after embryo transfer, while the clinical pregnancy rate was defined as a positive ultrasound visualization of the gestational sac at six weeks of pregnancy with the presence of fetal heart action after embryo transfer.

Statistical analysis was conducted using IBM SPSS Statistics for Windows, Version 22 (Released 2013; IBM Corp., Armonk, New York, United States)​​​​​. The Shapiro-Wilk test was used to assess the normality of variable distributions. For continuous independent variables with a normal distribution, means and standard deviations were calculated, while medians and interquartile ranges (IQRs) were used for those without a normal distribution. Student’s t-test was used to determine the significance of differences for normally distributed variables, whereas the Mann-Whitney U-test was used for variables that did not follow a normal distribution. A p-value of <0.05 was considered statistically significant.

## Results

In the period 2015-2017 year a study including 400 patients was conducted in the Clinic for Women's Diseases and Childbirth in Petrova. The patients were divided into two groups: the control group and the study group. The control group enrolled 300 patients, while the study group enrolled 100 patients. The study group was further divided into two subgroups: the subgroup of normal responders, which included 85 patients, and the subgroup of high responders, which included 15 patients.

The concentration of E2 and P4 in subjects of the control and test groups was analyzed. A significantly higher concentration of E2 was found on the day of hCG administration in the study group as compared to the control group (P<0.001). Similarly, a significantly higher P4 concentration was found on the day of hCG administration and on day 5 in the study group as compared to the control group (P<0.001) (Table 1). P4 and E2 concentrations on the day of hCG administration were also positively correlated (rS=0.578; P<0.001). Likewise, a positive correlation between P4 concentration on the fifth day of the cycle and E2 concentration on the day of hCG administration was demonstrated (rS=0.480; P<0.001). The mean value of P4 on the fifth day of the control group was also determined, which is 315 nmol/L, considering that the mentioned value is the discrimination limit on the basis of which the patients in the study group were classified for BET or FET transfer of embryos.

**Table 1:** Concentration of E2 and P4 in test subjects of the control and test groups C: median; 25. P.: 25. percentile; 75. P.: 75. percentile; Z: value of Mann-Whitney test: P: statistical significance; P4: progesterone

Variable	Group of patients		Statistical significance	
	Control C (25. P-75. P)	Study C (25. P-75. P)	Z	P
E2 concentration on hCG day	1560.75 (943.06-2200.00)	2850.86 (2285.60-3922.18)	9.722	<0.001
P4 concentration on hCG day	2.10 (1.60-2.80)	5.45 (5.20-6.30)	14.986	<0.001
P4 concentration on the fifth cycle day	312.35 (253.95-364.50)	312.80 (303.10-485.80)	4.617	<0.001

The number of retrieved oocytes, the number of fertilized oocytes, and the number of transferred embryos between the control and test groups were analyzed (Table 2). Subjects in the study group had a higher number of retrieved oocytes, a higher number of fertilized oocytes (P< 0.001), and an equal number of transferred embryos as compared to the control group (P=0.631).

**Table 2:** Number of retrieved oocytes, number of fertilized oocytes and number of transferred embryos in the control and study group C: median; 25. P.: 25. percentile; 75. P.: 75. percentile; Z: value of Mann-Whitney test; P: statistical significance

Variable	Group of patients		Statistical significance	
	Control C (25. P-75. P)	Study C (25. P-75. P)	Z	P
Number of retrieved oocytes (N)	7 (5-11)	14 (10-17)	8.853	<0.001
Number of fertilized oocytes (N)	5 (3-8)	9.5 (7-12)	7.993	<0.001
Number of transferred embryos (N)	2 (1-2)	2 (1-2)	0.481	0.631

The relationship between the concentration of P4 on the day of hCG administration and the number of retrieved oocytes was also analyzed. In the total sample, a statistically significant correlation was found between the concentration of P4 on the day of hCG administration and the number of retrieved oocytes (rS=0.540, P<0.001).

In the control group, the correlation coefficient was rS=0.415; P<0.001, while in the study group, when normal and high responders are observed together, no connection was found. Also, a similar result was found for the number of fertilized oocytes and P4 concentration on the day of hCG administration; in the control group, the correlation coefficient was rS=0.360; P<0.001, while in the study group, the association was absent.

In the group of normal responders, no statistically significant correlation was found between the concentration of P4 on the day of hCG administration and the number of retrieved oocytes (rS=-0.140, P=0.202), while in the group of high responders, a statistically significant correlation was found between the concentration of P4 on the day of administration of hCG and the number of retrieved oocytes (rS=0.628, P=0.012).

A statistically significant correlation between the concentration of P4 on the fifth day of the cycle and the number of retrieved oocytes was shown in all subjects. The higher the concentration, the higher the number of retrieved oocytes was seen (rS=0.455; P<0.001). Furthermore, a correlation was found between the concentration of P4 on the fifth day of the cycle and the number of retrieved oocytes in the group of high responders (rS=0.617; P=0.014).

A higher P4/E2 concentration ratio was found on the day of hCG administration in the study group as compared to the control group (P<0.001). Likewise, a similar result was obtained by analyzing the ratio of P4 concentration on the day of hCG administration/number of retrieved oocytes between the two groups (P<0.001) (Table 3). In the control group, BET was performed, while in the study group, BET was performed in 58% of cases and cryopreserved embryo transfer in the remaining 42%, which is statistically significant (χ^2^=140.782; P<0.001).

**Table 3:** P4/E2 ratio on the day of hCG administration and P4 concentration ratio on the hCG day/number of retrieved oocytes in all subjects C: median; 25. P.: 25. percentile; 75. P.: 75. percentile; Z: value of Mann-Whitney test; P: statistical significance; P4: progesterone

Variable	Group of patients		Statistical significance	
	Control C (25. P-75. P)	Study C (25. P-75. P)	Z	P
P4/E2 ratio on hCG day	0.430 (0.320-0.595)	0.590 (0.485-0.800)	6.784	<0.001
P4 concentration ratio on the hCG day/number of retrieved oocytes	0.280 (0.195-0.390)	0.420 (0.340-0.610)	8.247	<0.001

The conception rate in both groups of patients was about one-third of all procedures performed. Patients in the study group had a discretely better conception rate as compared to the control group (36.0% vs. 34.3%), but without statistical significance (P=0.762) (Table 4). The analysis between the groups was also done based on the clinical pregnancy rate. Table 4 shows the results of the clinical pregnancy rate in both groups of patients. It was shown that patients with elevated values ​​of preovulatory P4 had a higher frequency of clinical pregnancy rate as compared to the control group (32.0% vs. 26.0%), but without statistical significance (P=0.245).

**Table 4:** Conception rate and clinical pregnancy rate in the control and study group χ^2^=0.092; P=0.762: df=3; rS=0.455; χ^2^=1.354; P=0.245; df=3; rS=0.760

Group		Number	Conception rate		Clinical pregnancy rate		Total
			Yes	No	Yes	No	
Control	N (%)		103 (34.3)	197 (65.7)	78 (26.0)	222 (74.0)	300 (100.0)
Study	N (%)		36 (36.0)	64 (64.0)	32 (32.0)	68 (68.0)	100 (100.0)

The concentrations of E2 and P4 in the study group were also analyzed. A significantly higher concentration of E2 was found on the day of hCG administration in high responders as compared to normal responders (P<0.001). Likewise, a similar result was found for P4 concentrations on the day of hCG administration (P=0.006) and on day 5 of the cycle (P<0.001); hence, the high responders had a higher concentration of P4 on the day of hCG administration and P4 on the fifth day of the cycle as compared to the normal responders (Table 5).

**Table 5:** Concentration of E2 and P4 in the study group C: median; 25. P.: 25. percentile; 75. P.: 75. percentile; Z: value of Mann-Whitney test; P: statistical significance; P4: progesterone; hCG: human chorionic gonadotropin

Variable	Group of patients		Statistical significance	
	Normal responders C (25. P-75. P)	High responderis C (25. P-75. P)	Z	P
E2 concentration on hCG day	2722.00 (2121.00-3480.00)	4294.70 (3859.58-5862.60)	4.706	<0.001
P4 concentration on hCG day	5.40 (5.10-5.90)	6.40 (5.60-7.80)	2.758	0.006
P4 concentration on the fifth cycle day 5	311.10 (301.40-472.30)	492.60 (352.90-563.80)	3.697	<0.001

The number of retrieved oocytes, the number of fertilized oocytes, and the number of transferred embryos in the study group were analyzed. High responders had a higher number of retrieved oocytes (P<0.001), a higher number of fertilized oocytes (P=0.006), and an equal number of transferred embryos as compared to normal responders (P=0.590) (Table 6).

**Table 6:** The number of retrieved oocytes, the number of fertilized oocytes, and the number of transferred embryos in the study group C: median; 25. P.: 25. percentile; 75. P.: 75. percentile; Z: value of Mann-Whitney test; P: statistical significance

Variable	Group of patients		Statistical significance	
	Normal responders C (25. P-75. P)	High responders C (25. P-75. P)	Z	P
Number of retrieved oocytes (N)	13 (10-15)	20 (16-23)	4.615	<0.001
Number of fertilized oocytes (N)	9 (6-12)	11 (10-12)	2.764	0.006
Number of transferred embryos (N)	2 (1-2)	2 (1-2)	0.540	0.590

A higher P4/E2 concentration ratio was found on the day of hCG administration in normal responders as compared to high responders (P< 0.001). A similar result was obtained by analyzing the ratio of P4 concentration on the day of hCG administration/number of retrieved oocytes, where a higher ratio was found in normal responders as compared to high responders (P=0.004) (Table 7).

**Table 7:** P4/E2 concentration ratio on the day of hCG administration and P4 concentration ratio on the day of hCG administration a/b C: median; 25. P.: 25. percentile; 75. P.: 75. percentile; Z: value of Mann-Whitney test; P: statistical significance; P4: progesterone; hCG: human chorionic gonadotropin

Variable	Group of patients		Statistical significance	
	Normal responders C (25. P-75. P)	High responders C (25. P-75. P)	Z	P
P4/E2 ratio on hCG day	0.62 (0.50-0.84)	0.47 (0.42-0.50)	3.240	<0.001
P4 on hCG day a/b number of retrieved oocytes ratio	0.44 (0.36-0.68)	0.34 (0.29-0.40)	2.873	0.004

The conception rate in the study group was also analyzed. High responders had a slightly better conception rate as compared to normal responders (40.0% vs. 35.3%), but without statistical significance (P=0.469) (Table 8). Also, the clinical pregnancy rate in the study group was analyzed. Table 8 shows the clinical pregnancy rates in normal responders and high responders. In the group of high responders, a slightly better rate of clinically confirmed pregnancies was recorded as compared to normal responders (40.0% vs. 30.6%), but without statistical significance (P=0.330).

**Table 8:** Conception rate and clinical pregnancy rate in the study group χ^2^=0.726; P=0.469; df=3; rS=0.455; χ^2^=0.471; P=0.330; df=3; rS=0.480

Stimulation response		Number	Conception rate		Clinical pregnancy rate		Total
			Yes	No	Yes	No	
Normal responders	N (%)		30 (35.3)	55 (64.7)	26 (30.6)	59 (69.4)	85 (100.0)
High responders	N (%)		6 (40.0)	9 (60.0)	6 (40.0)	9 (60.0)	15 (100.0)

An analysis of the type of transfer in the study group was also made. In the group of normal responders, 34 FET transfers (40.0%) were made at postovulatory P4 values ​​>315 nmol/L and 51 BET transfers at postovulatory P4 values ​​<315 nmol/L (60.0%), while in the high responders, eight (53.3%) FET and seven (46.7%) BET transfers were made by randomization (Table 9).

**Table 9:** Type of transfer in the study group FET: frozen/thawed embryo transfer; BET: blastocyst embryo transfer

Group of patients	Number	Type of transfer		Total
		FET	BET	
Normal responders	N (%)	34 (40.0)	51 (60.0)	85 (100.0)
High responders	N (%)	8 (53.3)	7 (46.7)	15 (100.0)

Table 10 shows the conception rate for the study group regarding the type of transfer. There was a statistically significant difference, i.e., a higher rate of conception rate was seen with the FET transfer (P=0.032), which is clinically meaningful. An analysis of the clinical pregnancy rate in the study group was also made with regard to the type of transfer. It was shown that the clinical pregnancy rate was higher with the FET transfer as compared to the BET transfer (40.5% vs. 25.9%), but with marginal statistical significance (P=0.092).

**Table 10:** Conception rate and clinical pregnancy rate in the study group with regard to the type of transfer FET: frozen/thawed embryo transfer; BET: blastocyst embryo transfer; χ^2^=4.243; P=0.032; df=3; rS=0.578; χ^2^=2.391; P=0.092; df=3; rS=0.545

Type of transfer	Number	Conception rate		Clinical pregnancy rate		Total
		Yes	No	Yes	No	
FET	N (%)	20 (47.6)	22 (52.4)	17 (40.5)	25 (59.5)	42 (100.0)
BET	N (%)	16 (27.6)	42 (72.4)	15 (25.9)	43 (74.1)	58 (100.0)

Table 11 shows the conception rate in the normal responders regarding the type of transfer. There was a statistically significant difference, i.e., a higher conception rate was recorded with the FET transfer as compared to the BET transfer (50.0% vs. 25.5%, χ^2^=5.366; P=0.019). For normal responders, a higher clinical pregnancy rate was recorded with the FET transfer as compared to the BET transfer (41.2% vs. 23.5%) with marginal statistical significance (P=0.092).

**Table 11:** Conception rate and clinical pregnancy rate in normal responders regarding the type of transfer FET: frozen/thawed embryo transfer; BET: blastocyst embryo transfer; χ^2^=5.366; P=0.019; df=3; rS=0.575; χ^2^=2.391; P=0.092; df=3; rS=0.478

Type of transfer	Number	Conception rate		Clinical pregnancy rate		Total
		Yes	No	Yes	No	
FET	N (%)	17 (50.0)	17 (50.0)	14 (41.2)	20 (58.8)	34 (100.0)
BET	N (%)	13 (25.5)	38 (74.5)	12 (23.5)	39 (76.5)	51 (100.0)

An analysis of the conception rate and the clinical pregnancy among high responders was made with regard to the type of transfer. Table 12 shows the results of the aforementioned analysis. With the BET transfer, a discretely better conception rate was recorded as compared to the FET transfer (42.9% vs. 37.5%), but without statistical significance (P=0.622). For the same group, no statistical significance (P=0.622) was shown in the clinical pregnancy rate with regard to the type of transfer, although a slightly better clinical pregnancy rate was recorded with the BET transfer as compared to the FET transfer (42.9% against 37.5%).

**Table 12:** Conception rate and clinical pregnancy rate among high responders regarding the type of transfer FET: frozen/thawed embryo transfer; BET: blastocyst embryo transfer; χ^2^=0.045; P=0.622; df=3; rS=0.524

Type of transfer	Number	Conception rate		Clinical pregnancy rate		Total
		Yes	No	Yes	No	
FET	N (%)	3 (37.5)	5 (62.5)	3 (37.5)	5 (62.5)	8 (100.0)
BET	N (%)	3 (42.9)	4 (57.1)	3 (42.9)	4 (57.1)	7 (100.0)

## Discussion

The first paper on the influence of elevated preovulatory P4 was published by Feldberg et al. [20] in 1989. Their research showed a significantly lower rate of implantation and clinical pregnancies in the case of elevated preovulatory P4 as compared to the patients with normal values of preovulatory P4. Numerous studies, published later, talk about the unfavorable influence of elevated levels of preovulatory P4 on the outcome of IVF [13-15]. On the other hand, there are also studies that prove that there is no unfavorable influence of an elevated level of preovulatory P4 on the outcome of IVF [16,17]. Therefore, the appearance of an increase in preovulatory P4, as well as P4 values ​​in the serum during the mid-luteal phase, which can serve as a predictive factor for the outcome of the IVF procedure, represents a puzzle without clearly formed guidelines and strategies for the treatment of such patients.

Data from the United Kingdom for 2007 show that the success rate of IVF procedures in patients under 35 years of age is 32.3%, while in patients aged 40-42 years the success rate is only 11.9% [21]. Labarta et al. [22] analyzed the age of patients with elevated preovulatory P4 as compared to normal values. Their research showed that the patients, who had higher preovulatory P4 values, were older as compared to the patients with normal preovulatory P4 values ​​(27.5 ± 4.2 vs. 22 ± 2.2 years, P=0.017), with an average age of 24.7 ± 4.3 years. On the other hand, the results of our research showed that there was no difference in relation to the age of the subjects (P=0.096), with an average age of 34 years in both groups. Furthermore, when analyzing the difference in age between normal and high responders, it was not shown that there is a significant difference between them (P=0.540), with an average age of 34 years for normal responders and 32 years for high responders. When interpreting these results, it should be taken into account that their research included only 12 respondents; six each in the control and study groups; therefore, the difference in results compared to our research is understandable. Also, in their research, the patients in both groups were significantly younger as compared to the patients in our study, so this may also be the reason for the mentioned differences in the results.

In our study, patients in both groups were matched according to BMI (P=0.760), stimulation protocol, gonadotropin doses (P=0.447), and the number of transferred embryos (P=0.631). BMI analysis in normal and high responders has shown that there was no difference between the mentioned groups (P=0.209). Only nine patients had a body mass index greater than 30 kg/m^2^, of which only one subject had a BMI greater than 35 kg/m^2^. The administered dose of gonadotropin in both normal and high responders was 1800 IJ (P=0.486), and both groups were matched according to the number of transferred embryos (P=0.590).

Research has shown that both low E2 values ​​(500 pg/mL) and high E2 values ​​(>4,000 pg/mL) on the day of hCG result in a low or high number of retrieved oocytes, and therefore in a reduction in the possibility of realizing pregnancy. Elevated or decreased E2 values ​​lead to impaired maturation of oocyte cytoplasm, ultimately reducing the conception rate and clinical pregnancy rate [23]. Also, elevated E2 values ​​in the serum are directly related to an increased number of follicles and, according to some studies, to a premature increase in preovulatory P4 [24]. Our research has shown that E2 values ​​on the day of hCG administration were significantly higher in patients with elevated preovulatory P4 values ​​(P<0.001) (Table 4), and a positive correlation was also found between P4 and E2 concentrations on the day of hCG administration (rS=0.578; P<0.001). Requena et al. [19] also found a positive correlation between P4 and E2 concentrations on the day of hCG administration; E2 values ​​followed the increase of P4 values ​​(P<0.001). A significantly higher concentration of E2 was found on the day of hCG administration in high responders as compared to normal responders (P<0.001) (Table 12).

In recent years, the ratio of P4 and E2 on the day of hCG administration has been actualized in the scientific literature, given that the receptivity of the endometrium is determined by the complex interactions of P4 and E2. Younis et al. [25] as well as Arora et al. [26] found that the P4/E2 ratio is more accurate in detecting poor ovarian reserve and fewer oocytes obtained during aspiration. On the other hand, there are also studies that show that an increase in the P4/E2 ratio results in a higher number of retrieved oocytes and that it does not negatively affect the pregnancy rate in normo-ovulatory patients [15,27]. Our research confirmed the results of the latter studies, as it was shown that the P4/E2 ratio was higher in patients with elevated preovulatory P4 as compared to the control group (0.590 vs. 0.430, P<0.001) (Table 6). When the study group was divided into normal and high responders, it was shown that the P4/E2 ratio was higher in the normal responders (0.620 vs. 0.470, P<0.001) (Table 7). A higher ratio of P4 concentration on the day of hCG administration and the number of retrieved oocytes was found in the study group as compared to the control group (0.420 vs. 0.280, P<0.001) (Table 6). Furthermore, a higher ratio of P4 concentration on the day of hCG administration and the number of retrieved oocytes were found in normal responders as compared to high responders (0.440 vs. 0.340, P=0.004) (Table 7). Some authors went even further and analyzed the influence of the ratio of P4 concentration on the day of hCG administration and the number of retrieved oocytes on the outcome of the IVF procedure. A study by Aflatoonian et al. [28] found that the absolute value of preovulatory P4 and the P4/E2 ratio are not good predictors of the outcome of the IVF procedure. Furthermore, the ratio of P4 to the number of metaphase II oocytes was shown to be predictive of implantation rate and pregnancy rate, but had no effect on fertilization rate [28]. Singh et al. [29] showed that the ratio of P4/oocyte number on hCG day can be useful in predicting the outcome of the IVF procedure as compared to P4 values ​​alone. On the other hand, a recent study by Hill et al. [30] did not prove the advantage of using the ratio P4/number of oocytes on the day of hCG as a predictor of the outcome of the IVF procedure in relation to either the value of preovulatory P4 or the number of retrieved oocytes on the day of hCG and, on the outcome of the IVF procedure.

During the analysis of the number of retrieved oocytes, the number of fertilized eggs and the number of transferred embryos between both groups, it was shown that the patients in the study group had a higher number of retrieved oocytes, a higher number of fertilized eggs (P<0.001) and equally the number of transferred embryos as compared to the control group (P=0.631) (Table 5). High responders had a higher number of retrieved oocytes (P<0.001), a higher number of fertilized oocytes (P=0.006), and an equal number of transferred embryos as compared to normal responders (P=0.590). Furthermore, a statistically significant correlation was found between the concentration of P4 on the day of hCG administration and the number of retrieved oocytes in the total sample (rS=0.540, P<0.001). In the control group, the correlation coefficient was rS=0.415; P<0.001, while in the study group, when normal and high responders are observed together, no association was found. When normal and high responders were analyzed separately, a statistically significant correlation was found between the concentration of P4 on the day of hCG administration and the number of retrieved oocytes (rS=0.628, P=0.012) in high responders, while there was no correlation in normal responders (rS=-0.140, P=0.202). A statistically significant correlation between the concentration of P4 on the fifth day of the cycle and the number of retrieved oocytes was shown in all patients. The higher the concentration, the higher the number of retrieved oocytes (rS=0.455; P<0.001), and the same was shown in the group of high responders (rS=0.617; P=0.014). Li et al. [31] found that the number of retrieved oocytes was higher in patients with elevated preovulatory P4 values. Similarly, Bosch and colleagues [32] showed that the number of retrieved oocytes was higher in patients with elevated preovulatory P4 as compared to patients with normal preovulatory P4 ​​(P<0.0001). They also analyzed the correlation between E2 and P4 values ​​on the day of hCG in cases of elevated preovulatory P4, and also proved the correlation between E2 and P4 values; higher E2 values ​​were registered with an increase in preovulatory P4 (P<0.0001). It has been shown that elevated preovulatory P4 affects only the receptivity of the endometrium, changing the "implantation window", without affecting the quality of the embryos [33]. Our research has shown that a greater number of fertilized eggs were in the control group as compared to the study group (five fertilized oocytes as compared to seven retrieved on average in the control group, that is, 9.5 fertilized oocytes to 14 retrieved in the study group).

Similar results were obtained when analyzing normal and high responders; a higher number of fertilized eggs than the total number of retrieved oocytes was observed in normal responders, as compared to high responders (nine fertilized eggs as compared to 13 retrieved on average in normal responders, or 11 fertilized oocytes as compared to 20 retrieved in high responders). These results, contrary to the previously mentioned research, nevertheless indirectly have shown that elevated preovulatory P4 can have a potentially detrimental effect on the quality of fertilized eggs, because a higher number of fertilized oocytes was registered in the group with normal P4 values. Our results were confirmed by Huang et al. [34], who, in an analysis of 4236 IVF cycles, clearly showed a negative effect of elevated P4 ​​on hCG day on embryo quality, regardless of basal FSH values, total gonadotropin dose, women's age, or ovarian stimulation time. On the other hand, Griesinger et al. [18] and Requena et al. [19] did not find a negative effect of elevated preovulatory P4 ​​on the quality of either oocytes or embryos, which is contrary to the results of our research.

In the context of stimulated cycles, the potentially detrimental role of P4 during the follicular phase has been the focus of interest of many scientists and the subject of controversy in recent years. Elevated P4 ​​during the late follicular phase probably affects endometrial secretory changes, resulting in impaired endometrial receptivity. On the other hand, patients who respond adequately to controlled ovarian hyperstimulation (COH) are more likely to produce better oocytes. Therefore, the consequences of elevated preovulatory P4 on the clinical outcome of the pregnancy of patients with a good response to stimulation will depend on the balance of two antagonistic factors: the probability of obtaining more oocytes with a good ovarian response and impaired receptivity of the endometrium, which is conditioned by the premature exposure of the endometrium to P4 [19]. In their review article, Sonigo et al. [33] analyzed numerous studies and found conflicting views. One group of scientists claims that there is no negative effect of elevated preovulatory P4 ​​on the outcome of the IVF procedure, while others claim the complete opposite, that is, that there is a negative effect.

A large meta-analysis by Venetis et al. [15] with more than 60,000 cycles has shown that elevated preovulatory P4 is associated with a lower pregnancy rate. On the other hand, Huang et al. [35] have shown that elevated preovulatory P4 does not harm the clinical outcome of the IVF procedure. Requena et al. [19] have shown that there is a marginal statistical significance related to the influence of preovulatory P4 on the conception rate, with a value of P4>5.7 nmol/L (P=0.045) and on the clinical pregnancy rate at the same value of P4 (P=0.048) in the context of a strong ovarian response to stimulation. Our research has shown similar results. The conception rate was about one third of all procedures performed, with discretely better results in the study group (36.0% in the study group vs. 34.3% in the control group, χ^2^=0.092; P=0.762), but without statistical significance (Table 7). The same result was obtained when analyzing the conception rate in normal and high responders; the conception rate was about one-third in both groups of patients, with a slightly better conception rate in high responders (40.0% in high responders as compared to 35.3% in normal responders, χ^2^=0.726; P=0.469), but without statistical significance. Our results practically show that elevated preovulatory P4 has even somewhat more favorable effect on the conception rate as compared to normal values ​​of preovulatory P4. Furthermore, we have shown that high responders have a better conception rate as compared to normal responders, thereby confirming the research of Griesinger and colleagues [18]. In accordance with the assumption about the detrimental effect of elevated preovulatory P4, the influence of the type of transfer (FET or BET) on the outcome of the IVF procedure was also analyzed. Corti et al. [36] showed that BET cannot completely overcome the detrimental effect of elevated P4 ​​on the day of hCG on the outcome of the IVF procedure. However, Papanikolaou et al. [37] reported better IVF success with BET as compared to day 3 embryo transfer. Their opinion is shared by Huang et al. [35], who also showed that BET could improve the outcome with elevated P4 ​​on the day of hCG. Yang et al. [38] published the first and only prospective clinical study comparing cryopreserved embryo transfer with BET. FET cycles had a comparable rate of live births with BET (38.1% vs. 13.9%) at preovulatory P4=6 nmol/L, and this result is probably due to the small sample size in the study. On the other hand, Healy et al. [39] showed that elevated P4 on the day of hCG was negatively associated with the rate of live births during BET, but not during the FET procedure. Embryo cryopreservation and FET transfer in a natural cycle increase the rate of live births in cases of elevated preovulatory P4. Their opinion is shared by Wong et al. [40], who propose cryopreservation of all embryos, without BET, to optimize the success of the IVF procedure. In our research, in the study group, there was a higher proportion of positive conception rate during the FET procedure (47.6% vs. 27.6%, χ^2^=4.243; P=0.032). Furthermore, in the group of normal responders, there was a statistically significant difference in the frequency of the positive conception rate depending on the type of transfer (50.0% in the case of the FET procedure or 25.5% in the case of the BET procedure, χ^2^=5.366; P=0.019), contrary to the high responders in whom a better conceptio rate was recorded with the BET procedure (42.9% with the BET procedure as compared to 37.5% with the FET procedure, χ^2^=0.045; P=0.622), but without statistical significance. It has been shown that the BET procedure in normal responders with elevated postovulatory P4 (>315 nmol/L) adversely affects the pregnancy outcome; therefore, the conception rate could be improved using the FET procedure. On the other hand, this does not apply to high responders, where a better conception rate was recorded with the BET procedure, which confirmed the research of Griesinger and colleagues [18]. From the results of our research, it can be concluded that, in situations of elevated preovulatory P4, cryopreservation of all embryos and subsequent transfer in the natural cycle should be considered. In this way, the outcome of the IVF procedure in normal responders would be improved, while in high responders, the most common complication of COH, namely OHSS, would be mostly prevented, although with a slightly less favorable pregnancy rate.

The clinical pregnancy rate in the control and the study group was also analyzed. In both groups, the total percentage of clinical pregnancy rate was about one third of all performed procedures, although with a slightly better result in the study group, but without statistical significance (32.0% in the study group vs. 26.0% in the control group, χ^2^=1.354; P=0.245), and the same result was obtained when analyzing normal and high responders (40.0% in high responders as compared to 30.6% in normal responders, χ^2^=0.471; P=0.330). For the study group, a marginal statistical significance was shown in terms of clinical pregnancy rate with regard to the type of transfer (40.5% in the case of the FET procedure vs. 25.9% in the case of the BET procedure, χ^2^=2.391; P=0.092). For normal responders, marginal statistical significance was also shown in terms of the clinical pregnancy rate with regard to the type of transfer (41.2% in the case of the FET procedure vs. 23.5% in the case of the BET procedure, χ^2^=2.391; P=0.092), and the same also showed for high responders (42.9% with the BET procedure vs. 37.5% with the FET procedure, χ^2^=0.045; P=0.622). As our results show, elevated preovulatory P4 ​​has an even more favorable influence on the clinical pregnancy rate as compared to normal preovulatory P4. This practically means that, if a biochemical pregnancy or an early miscarriage occurs in patients with elevated preovulatory P4, it should not be viewed through the prism of P4 on the day of hCG, but there are also a number of other factors in patients that could influence such an outcome. However, when the type of transfer is taken into account, the results for normal and high responders are different. Why the clinical pregnancy rate is better in high responders is not yet clear in the literature. It is assumed that there are some factors in this group of patients due to which the outcome of IVF with the BET procedure is better. These factors potentially include endometrial receptivity and oocyte and embryo quality, although these assumptions need to be confirmed by prospective randomized studies [18]. On the other hand, the latest research by Lawrence and colleagues [41] showed that elevated preovulatory P4 has a negative effect on the outcome of the IVF procedure, both on the conception rate and on the clinical pregnancy rate.

Although we provided valuable insights into the effect of preovulatory P4 on the IVF outcome, our study had several limitations. First, the sample size was small compared to some other research (especially the high responders group, which was small with only 15 participants), limiting the findings' statistical power and generalizability. Additionally, this was a one-center study compared to multi-center studies, which could also affect the study results and conclusions of this research. Finally, the potential variability in assay measurement of P4 could also be a limitation in this study.

## Conclusions

Elevated values ​​of preovulatory P4 in all patients in the study group after stimulation of ovulation in the IVF procedure do not have an adverse effect on the success of the procedure, as seen through the conception rate and the clinical pregnancy rate. Similarly, elevated postovulatory P4 (>315 nmol/L) in the study group does not have an adverse effect on the outcome of IVF pregnancy in normal responders with blastocyst cryopreservation/thawing and transfer in a natural cycle, and in all high responders with transfer of fresh (BET) or cryopreserved/thawed embryos. Despite the increased value of preovulatory P4 in the patients in the study group, there is no adverse effect on the outcome of pregnancy after the IVF procedure as compared to the patients in the control group with lower concentrations of preovulatory P4. In the study group of patients with elevated preovulatory P4, the pregnancy outcome is better in normal responders with higher concentrations of postovulatory P4 (>315 nmol/L) in whom, after cryopreservation/thawing, BET was performed in a natural cycle as compared to normal responders with lower concentrations of postovulatory P4 (<315 nmol/L) in whom fresh BET was performed in the same cycle. In the study group of patients with randomized embryo transfer in high responders with elevated preovulatory P4 and postovulatory P4 (>315 nmol/L), the success of the IVF procedure is emphasized more in cases of transfer of fresh blastocysts in the same cycle (BET), in relation to embryo cryopreservation/thawing and transfer in the natural cycle.

## References

[1] McCracken JA, Custer EE, Lamsa JC (1999). Luteolysis: a neuroendocrine-mediated event. Physiol Rev.

[2] De Geyter C, De Geyter M, Huber PR, Nieschlag E, Holzgreve W (2002). Progesterone serum levels during the follicular phase of the menstrual cycle originate from the crosstalk between the ovaries and the adrenal cortex. Hum Reprod.

[3] Keck C, Neulen J, Breig-Lauel S, Breckwoldt M (1999). Elevated serum progesterone concentrations during the early follicular phase of the menstrual cycle: clinical significance and therapeutic implications. Gynecol Endocrinol.

[4] Sims JA, Seltman HJ, Muasher SJ (1994). Early follicular rise of serum progesterone concentration in response to a flare-up effect of gonadotrophin-releasing hormone agonist impairs follicular recruitment for in-vitro fertilization. Hum Reprod.

[5] Tang Y, Gong F, Lin G, Lu G (2007). Early follicular progesterone concentrations and in vitro fertilization pregnancy outcomes. Fertil Steril.

[6] Huang JC, Jackson KV, Hornstein MD, Ginsburg ES (1996). The effect of elevated serum progesterone during ovulation induction in in vitro fertilization-embryo transfer. J Assist Reprod Genet.

[7] Kolibianakis EM, Zikopoulos K, Smitz J, Camus M, Tournaye H, Van Steirteghem AC, Devroey P (2004). Elevated progesterone at initiation of stimulation is associated with a lower ongoing pregnancy rate after IVF using GnRH antagonists. Hum Reprod.

[8] Younis JS (2011). "Premature luteinization" in the era of GnRH analogue protocols: time to reconsider. J Assist Reprod Genet.

[9] Kasum M, Radakovic B, Simunic V, Oreškovic S (2013). Preovulatory progesterone rise during ovarian stimulation for IVF. Gynecol Endocrinol.

[10] Kasum M, Simunić V, Vrčić H, Stanić P, Orešković S, Beketić-Orešković L (2014). Follicular progesterone elevations with ovulation induction for IVF. Gynecol Endocrinol.

[11] Silverberg KM, Martin M, Olive DL, Burns WN, Schenken RS (1994). Elevated serum progesterone levels on the day of human chorionic gonadotropin administration in in vitro fertilization cycles do not adversely affect embryo quality. Fertil Steril.

[12] Ubaldi F, Albano C, Peukert M (1996). Subtle progesterone rise after the administration of the gonadotrophin-releasing hormone antagonist cetrorelix in intracytoplasmic sperm injection cycles. Hum Reprod.

[13] Venetis CA, Kolibianakis EM, Papanikolaou E, Bontis J, Devroey P, Tarlatzis BC (2007). Is progesterone elevation on the day of human chorionic gonadotrophin administration associated with the probability of pregnancy in in vitro fertilization? A systematic review and meta-analysis. Hum Reprod Update.

[14] Kolibianakis EM, Venetis CA, Bontis J, Tarlatzis BC (2012). Significantly lower pregnancy rates in the presence of progesterone elevation in patients treated with GnRH antagonists and gonadotrophins: a systematic review and meta-analysis. Curr Pharm Biotechnol.

[15] Venetis CA, Kolibianakis EM, Bosdou JK, Tarlatzis BC (2013). Progesterone elevation and probability of pregnancy after IVF: a systematic review and meta-analysis of over 60 000 cycles. Hum Reprod Update.

[16] Martinez F, Coroleu B, Clua E (2004). Serum progesterone concentrations on the day of HCG administration cannot predict pregnancy in assisted reproduction cycles. Reprod Biomed Online.

[17] Yding Andersen C, Bungum L, Nyboe Andersen A, Humaidan P (2011). Preovulatory progesterone concentration associates significantly to follicle number and LH concentration but not to pregnancy rate. Reprod Biomed Online.

[18] Griesinger G, Mannaerts B, Andersen CY, Witjes H, Kolibianakis EM, Gordon K (2013). Progesterone elevation does not compromise pregnancy rates in high responders: a pooled analysis of in vitro fertilization patients treated with recombinant follicle-stimulating hormone/gonadotropin-releasing hormone antagonist in six trials. Fertil Steril.

[19] Requena A, Cruz M, Bosch E, Meseguer M, García-Velasco JA (2014). High progesterone levels in women with high ovarian response do not affect clinical outcomes: a retrospective cohort study. Reprod Biol Endocrinol.

[20] Feldberg D, Goldman GA, Ashkenazi J, Dicker D, Shelef M, Goldman JA (1989). The impact of high progesterone levels in the follicular phase of in vitro fertilization (IVF) cycles: a comparative study. J In Vitro Fert Embryo Transf.

[21] Cooke L, Nelson SM (2011). Reproductive ageing and fertility in an ageing population. Obstet Gynaecol.

[22] Labarta E, Martínez-Conejero JA, Alamá P, Horcajadas JA, Pellicer A, Simón C, Bosch E (2011). Endometrial receptivity is affected in women with high circulating progesterone levels at the end of the follicular phase: a functional genomics analysis. Hum Reprod.

[23] Papageorgiou T, Guibert J, Goffinet F, Patrat C, Fulla Y, Janssens Y, Zorn JR (2002). Percentile curves of serum estradiol levels during controlled ovarian stimulation in 905 cycles stimulated with recombinant FSH show that high estradiol is not detrimental to IVF outcome. Hum Reprod.

[24] Kyrou D, Al-Azemi M, Papanikolaou EG, Donoso P, Tziomalos K, Devroey P, Fatemi HM (2012). The relationship of premature progesterone rise with serum estradiol levels and number of follicles in GnRH antagonist/recombinant FSH-stimulated cycles. Eur J Obstet Gynecol Reprod Biol.

[25] Younis JS, Haddad S, Matilsky M, Ben-Ami M (1998). Premature luteinization: could it be an early manifestation of low ovarian reserve?. Fertil Steril.

[26] Arora R, Chan C, Ye XY, Greenblatt EM (2017). Progesterone, progesterone/estradiol and ART outcomes in day-5 transfer cycles. Gynecol Endocrinol.

[27] Lai TH, Lee FK, Lin TK, Horng SG, Chen SC, Chen YH, Wang PC (2009). An increased serum progesterone-to-estradiol ratio on the day of human chorionic gonadotropin administration does not have a negative impact on clinical pregnancy rate in women with normal ovarian reserve treated with a long gonadotropin releasing hormone agonist protocol. Fertil Steril.

[28] Aflatoonian A, Davar R, Hojjat F (2014). Elevated serum progesterone/ MII oocyte ratio on the day of human chorionic gonadotropin administration can predict impaired endometrial receptivity. Iran J Reprod Med.

[29] Singh N, Malik N, Malhotra N, Vanamail P, Gupta M (2016). Impact of progesterone (on hCG day)/oocyte ratio on pregnancy outcome in long agonist non donor fresh IVF/ICSI cycles. Taiwan J Obstet Gynecol.

[30] Hill MJ, Healy MW, Richter KS (2017). Revisiting the progesterone to oocyte ratio. Fertil Steril.

[31] Li PF, Zhu H, Tan L (2015). Effects of high progesterone on outcomes of in vitro fertilization-embryo transfer in patients with different ovarian responses. Syst Biol Reprod Med.

[32] Bosch E, Labarta E, Crespo J, Simón C, Remohí J, Jenkins J, Pellicer A (2010). Circulating progesterone levels and ongoing pregnancy rates in controlled ovarian stimulation cycles for in vitro fertilization: analysis of over 4000 cycles. Hum Reprod.

[33] Sonigo C, Dray G, Roche C, Cédrin-Durnerin I, Hugues JN (2014). Impact of high serum progesterone during the late follicular phase on IVF outcome. Reprod Biomed Online.

[34] Huang B, Ren X, Wu L (2016). Elevated progesterone levels on the day of oocyte maturation may affect top quality embryo IVF cycles. PLoS One.

[35] Huang PC, Chen MJ, Guu HF (2015). Effect of premature serum progesterone rise on embryo transfer outcomes and the role of blastocyst culture and transfer in assisted reproductive technology cycles with premature progesterone rise. Taiwan J Obstet Gynecol.

[36] Corti L, Papaleo E, Pagliardini L (2013). Fresh blastocyst transfer as a clinical approach to overcome the detrimental effect of progesterone elevation at hCG triggering: a strategy in the context of the Italian law. Eur J Obstet Gynecol Reprod Biol.

[37] Papanikolaou EG, Kolibianakis EM, Pozzobon C (2009). Progesterone rise on the day of human chorionic gonadotropin administration impairs pregnancy outcome in day 3 single-embryo transfer, while has no effect on day 5 single blastocyst transfer. Fertil Steril.

[38] Yang S, Pang T, Li R (2015). The individualized choice of embryo transfer timing for patients with elevated serum progesterone level on the HCG day in IVF/ICSI cycles: a prospective randomized clinical study. Gynecol Endocrinol.

[39] Healy MW, Patounakis G, Connell MT, Devine K, DeCherney AH, Levy MJ, Hill MJ (2016). Does a frozen embryo transfer ameliorate the effect of elevated progesterone seen in fresh transfer cycles?. Fertil Steril.

[40] Wong KM, Mastenbroek S, Repping S (2014). Cryopreservation of human embryos and its contribution to in vitro fertilization success rates. Fertil Steril.

[41] Lawrenz B, Melado L, Fatemi H (2018). Premature progesterone rise in ART-cycles. Reprod Biol.

